# Supplementary material for: A network dynamical simulation model for the study of antibiotic resistance in microbial communities
Source: Gut Microbes. 2026 Sep 19;18(1):2734703. doi: 10.1080/19490976.2026.2734703 (PMC13596919; doi:10.1080/19490976.2026.2734703)
Supplement: Supplementary file 1 [file KGMI_A_2734703_SM9053.zip › supplementary_file_1/Figures/ba_delta_gc_size_n1000.pdf]

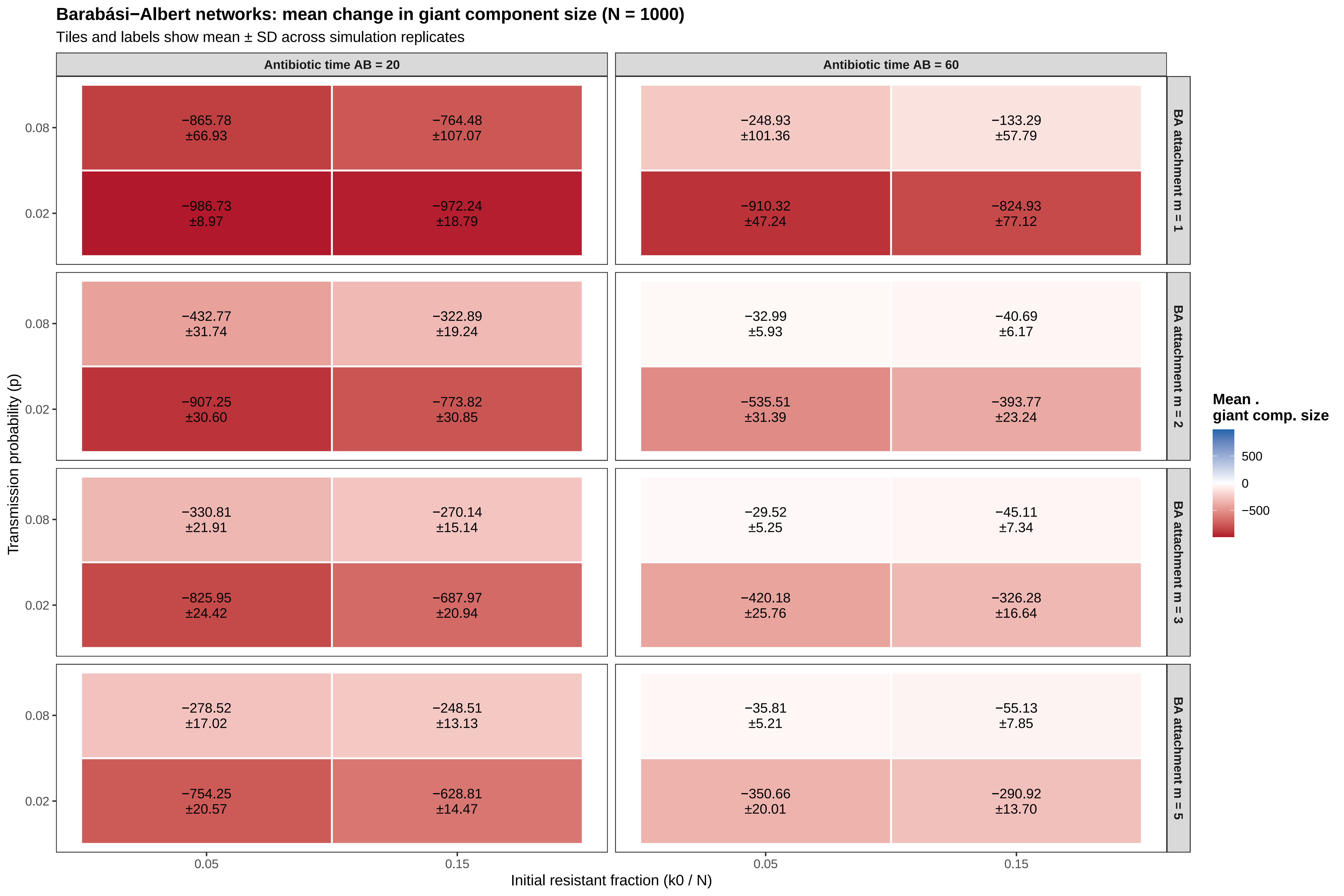

Barabási−Albert networks: mean change in giant component size (N = 1000)
Tiles and labels show mean ± SD across simulation replicates
Antibiotic time AB = 20
Antibiotic time AB = 60
BA attachment m = 1
BA attachment m = 2
BA attachment m = 3
BA attachment m = 5
−865.78 ±66.93
−764.48 ±107.07
−986.73 ±8.97
−972.24 ±18.79
−248.93 ±101.36
−133.29 ±57.79
−910.32 ±47.24
−824.93 ±77.12
−432.77 ±31.74
−322.89 ±19.24
−907.25 ±30.60
−773.82 ±30.85
−32.99 ±5.93
−40.69 ±6.17
−535.51 ±31.39
−393.77 ±23.24
−330.81 ±21.91
−270.14 ±15.14
−825.95 ±24.42
−687.97 ±20.94
−29.52 ±5.25
−45.11 ±7.34
−420.18 ±25.76
−326.28 ±16.64
−278.52 ±17.02
−248.51 ±13.13
−754.25 ±20.57
−628.81 ±14.47
−35.81 ±5.21
−55.13 ±7.85
−350.66 ±20.01
−290.92 ±13.70
Transmission probability (p)
0.08
0.02
Initial resistant fraction (k0 / N)
0.05
0.15
Mean .
giant comp. size
500
0
−500
